# A novel liquid-liquid cfDNA extraction method for targeted sequencing with colorectal cancer patient samples: a pilot study

**DOI:** 10.3389/fonc.2026.1812135

**Published:** 2026-04-29

**Authors:** Cheuk Yiu Tenny Chung, Allen Chi-Shing Yu, Kit Cheung, Chau-Ming Kan, Ken Hung On Yu, Cheuk Yin Lam, Daniel W. Bradbury, Vasu Saini, Kar Kee Tse, Mei Wai Fung, Xiao Meng Pei, Sze Chuen Cesar Wong, Peng Yin, Aldrin Kay-Yuen Yim, Garrett Lee Mosley, Ricky Y. T. Chiu

**Affiliations:** 1 Phase Scientific International Limited, Hong Kong, Hong Kong SAR, China; 2 Codex Genetics Limited, Hong Kong, Hong Kong SAR, China; 3 Department of Applied Biology and Chemical Technology, The Hong Kong Polytechnic University, Hong Kong, Hong Kong SAR, China

**Keywords:** cancer, cell-free DNA, liquid biopsy, liquid-liquid extraction, targeted sequencing

## Abstract

**Introduction:**

Cell-free DNA (cfDNA) in the bloodstream released from tumor, also known as circulating tumor DNA (ctDNA), has emerged as a promising biomarker for cancer detection and monitoring. Extraction of ctDNA from plasma liquid biopsies is an emerging approach with minimal invasiveness to detect gene mutations associated with colorectal cancer. One hurdle to ctDNA mutational analyses is obtaining sufficient quantity and good quality of DNA which is compatible with downstream diagnostic testing, especially when testing samples with limited volumes of plasma.

**Methods:**

We developed and evaluated a novel liquid-liquid extraction method optimized to concentrate and purify ctDNA from plasma samples. Analytical performance was assessed using reference materials and compared to a gold standard commercially available extraction kit under targeted panel next-generation sequencing. A pilot study was conducted on plasma samples from 16 colorectal cancer patients. Additionally, an independent, blinded external validation study was included to confirm the analytical validity of this workflow.

**Results:**

In analytical testing using reference materials, the novel extraction kit achieved higher DNA yield and higher tumor mutation detection rate compared to a gold standard commercially available extraction kit under targeted panel next-generation sequencing. In the pilot study of 16 colorectal cancer patient samples using this workflow with the liquid-liquid extraction method, an average of 73.4% (262/357) of the tumor mutations could be detected in low volumes of plasma, which is higher than the 50% pooled tumor mutation detection rate of plasma cfDNA in a previous meta-analysis. Parallel testing of plasma and tumor samples can enhance the detection of potentially actionable genes. Meanwhile, the workflow achieved full concordance across all samples, providing objective, third-party verification of its diagnostic precision and sensitivity.

**Discussion:**

These results demonstrate the promising performance of integrating liquid-liquid plasma extraction with targeted panel next-generation sequencing to identify cancer-associated mutations in colorectal cancer (CRC) patients.

## 1 Introduction

In the field of oncology diagnosis and management, evaluating patients’ genetic profiles and identifying oncogenic mutations is critical for developing a personalized treatment plan and predicting therapy outcomes. For example, identification of mutations is crucial for selecting the appropriate neoadjuvant chemotherapy drug to reduce the size of tumor before surgery (1, 2). Currently, tumor tissue biopsy is still preferred over blood for detecting mutation profiles for patients according to the NCCN Clinical Practice Guidelines in Oncology for multiple cancer types including lung, pancreatic, and colon cancer (3–5). However, there are several limitations, risks, and challenges associated with solid tumor biopsy-based analysis. First, collection of solid tumor tissues is an invasive procedure, and it is not always feasible to be collected for molecular testing. Second, the small pieces of tumor tissue collected may not accurately represent the overall genetic profile of the tumor due to tumor heterogeneity (6). Lastly, the conventional tissue biopsy approach has an inherent risk of tumor cell seeding along the needle track into the adjacent healthy tissue and circulatory system, leading to tumor cell dissemination and metastasis (7–9). In contrast, blood-based liquid biopsy sampling is a minimally invasive alternative approach, which does not disrupt the intact tumor structure, mitigates the risk of tissue biopsy-induced tumor metastasis, and can provide a more wholistic genetic profile assessment of the tumor. Previous studies have demonstrated the feasibility of detecting somatic mutations in cancer using plasma liquid biopsy samples, but the tumor tissue mutation detection rate of liquid biopsy was relatively low. The rate of using plasma to detect cancer mutation in tumor tissue varies from 22% to 85%, with an average of 50% among 12 different studies included in a meta-analysis (10). More recently, efforts have been made to improve the tumor mutation detection rate of sequencing based liquid biopsy testing by optimizing the sequencing methods or bioinformatics analysis (11). However, effective extraction of ctDNA from plasma is essential for highly sensitive detection of tumor mutation, and it is a critical challenge especially when limited plasma volumes are available in general clinical workflow.

Most commercially available kits for DNA extraction are solely based on solid phase extraction mechanisms, where lysed samples are directly added to silica columns or magnetic beads. The binding efficiency of the target DNA to the solid phase could be affected by the low concentration of target DNA and other interfering substances in the sample matrix. This direct solid-phase approach leaves open the possibility for incomplete binding or unbinding of the target DNA. To improve the binding efficiency, we utilize the liquid-liquid extraction process to purify and concentrate target DNA in a small liquid phase, while partitioning other biomolecules and contaminants to the opposite liquid phase, before final recovery steps.

Previously, in collaboration with MD Anderson, we investigated the use of liquid-liquid extraction to purify and concentrate cfDNA from bio-banked cancer patient plasma samples. Using ddPCR, the liquid-liquid extraction method demonstrated a 60% increase in DNA yield and 171% increase in mutant copy recovery when compared to the commercially available gold standard direct solid phase extraction platform, QIAamp Circulating Nucleic Acid Kit (QCNA) (12). In the present study, we evaluated the novel liquid-liquid extraction system’s application in targeted next generation sequencing-based testing and demonstrates the potential for enhanced analytical sensitivity in detecting low-frequency variant alleles in cfDNA compared to QCNA when using reference materials. Furthermore, in a pilot study of 16 colorectal cancer patients, we compared the somatic mutations detected between the paired solid tumor tissue and liquid biopsy samples processed with the liquid-liquid extraction system.

## 2 Materials and methods

### 2.1 Preparation and extraction of reference materials

Seraseq ctDNA Reference Material v2 containing known mutations with 1% and 0% (SeraCare Life Sciences) of variant allele frequencies (VAF) were mixed together in one to one ratio to prepare reference material with 0.5% of VAF. 1ml of 0.5% VAF reference material was extracted by PHASIFY MAX plasma cfDNA extraction kit (Phase Scientific International Limited) and QIAamp Circulating Nucleic Acid kit (QIAGEN), respectively, following the manufacturer’s protocol.

### 2.2 Patients and specimen collection

The study was conducted in accordance with the Declaration of Helsinki and approved by the Institutional Review Board (or Ethics Committee) of the Chinese University of Hong Kong-New Territories East Cluster (CUHK-NTEC CREC) (protocol code 2019.542, approved on 2019 Dec 3). A cohort of 16 patients with CRC were retrospectively retrieved from the biobank at Hong Kong Polytechnic University, following approval from the Ethics Committee (Ref: 2019.542) (13). All participants provided written informed consent prior to sample collection. To minimize confounding factors, only unrelated patients with histologically confirmed colon adenocarcinoma were included, while subjects with hereditary CRC or inflammatory bowel disorder were excluded. Tissue specimens were immediately preserved in RNAlater (Thermo Fisher) at 4 °C for 24 hours to stabilize both DNA and RNA, followed by storage at −80 °C. Peripheral blood samples were collected in K2EDTA anticoagulant tubes (Greiner Bio-One) tubes alongside the surgically resected tumor tissues, followed by plasma isolation using centrifugation at 1,600 rcf for 10 minutes and 16,000 rcf for 10 minutes at 4 °C. Plasma aliquots were flash-frozen and stored at −80 °C.

### 2.3 DNA isolation from tissue sample

The tissue samples were handled as previously described (13). Briefly, the tumor tissues were first shredded by a homogenizer and then extracted with the AllPrep DNA/RNA kit (Qiagen) following the manufacturer’s instructions.

### 2.4 DNA extraction from plasma sample

cfDNA extraction from 2 mL of plasma was performed using a PHASIFY MAX plasma cfDNA extraction kit (Phase Scientific International Limited) following the manufacturer’s protocol. Briefly, 2 mL of plasma was mixed with protein digestion reagents supplied by the kit and incubated at 37 °C for 15 min. The plasma mixture was then added to the provided first liquid two-phase solution and vortexed. The mixture was centrifuged for 6 min at 2,300 rcf to engage phase separation. The bottom phase was then manually extracted via pipette and added to a provided second liquid two-phase solution. The second liquid two-phase solution was vortexed and centrifuged for 1 min at 7,000 rcf. Then around 120 µL of the top phase was similarly extracted and added to an empty 2 mL micro-centrifuge tube. The extract was mixed with the kit-provided binding buffer solution and allowed to incubate together with kit-provided magnetic beads for 5 min. The mixture was then allowed to stand in magnetic racks for 5 min. The supernatant was then removed, and the bead was washed two separate times with kit-provided washing buffer. After removal of the 70% ethanol, the DNA pellet was dried at room temperature for 7 min and resuspended in 40 µL of kit-provided elution buffer.

### 2.5 DNA quantification

Concentration was assessed using a Qubit 4.0 Fluorometer (Thermo Fisher Scientific) with a Qubit™ dsDNA HS Assay Kit (Thermo Fisher Scientific) before sample storage at −20 °C.

### 2.6 Library preparation and targeted sequencing

TSO500 is an NGS assay that enables the comprehensive genomic profiling of tumor samples. TruSight Oncology 500 target panel (illumina) was used to detect mutations and identify other relative pan-cancer genes in the tumor samples. The generation of library preparations were performed according to the manufacturers’ instructions. Target sequencing was carried out using Illumina NextSeq2000 according to the manufacturer’s instructions. The Binary Base Call (BCL) files were converted to FASTQ files using the Illumina BCL Convert (v3.7.5).

### 2.7 Somatic mutation calling and bioinformatics analysis

The variant calling and interpretation were performed by Codex CoGenesis platform, which comprises high-performance and validated pipelines deployed on the Amazon Web Services. In brief, DNA sequencing data was aligned to the human reference genome (hg38) using the DRAGEN Bio-IT Platform (version 3.8.4, Illumina), followed by mutation calling using DRAGEN’s high-performance algorithm and default hard filters tailored for tumor-only analysis (14). Parameters recommended in the DRAGEN user manual was adopted to fine-tune the variant calling parameters, such as the use of “vc-enable-umi-solid” parameter for solid tumor samples and the vc-enable-umi-liquid parameter for plasma samples. An in-house panel of normals (PON) derived from 200 tumor-adjacent normal samples were used to mitigate potential sequencing artefacts associated with the Il-lumina TSO500 data.

Post-processing of the somatic mutations in variant call format (VCF) began with annotation using the Variant Effect Predictor (version 111_GRCh38, Ensembl) and vcfanno (version 0.3.4) (15). Over 150 population frequency and functional prediction meta data from multiple databases, including ClinVar (20240317) (16), gnomAD exomes and genomes (version 4.0), dbSNP (build 156), and dbNSFP (version 4.7c) (17) were added to the mutation calls. The annotated VCF was subsequently converted to Mutation Annotation Format (MAF) using vcf2maf (version 1.6.22).

To ensure the accuracy of the somatic mutation calls and eliminate potential sequencing artifacts and noncoding variants, the resulting MAF file was filtered using a custom Python script. Mutations were retained based on the following criteria: (1) tumor minor allele frequency (MAF) ≥ 0.05 for tissue samples or MAF ≥ 0.001 for plasma samples; (2) DRAGEN hard filter status of “PASS”; (3) exclusion of mutations flagged by the PON; (4) population allele frequencies < 0.001 according to gnomAD exome and genome; (5) exclusion of ClinVar variants reported as “benign,” “not_provided,” or “uncertain_significance” and germline in origin; (5) removal of noncoding variants except splice regions and promoter regions. This stringent filtering criteria set allowed us to focus the analysis on high-confidence, functionally relevant somatic mutations.

Interactive exploration and visualization of mutation call summary charts were generated on the Codex CoGenesis dashboard platform and maftools. Statistical analysis was performed using custom Python and R scripts.

### 2.8 External quality assessment by EMQN

Three blinded plasma samples were retrieved from European Molecular Genetics Quality Network (EMQN) for testing (Scheme: Lung Cancer (NSCLC) [Plasma] 2022; Lab ID: 3059). The plasma samples were extracted using PHASIFY MAX kit prior to library preparation and target capture sequencing using TSO500 panel and Illumina NextSeq 2000. The variant calling and interpretation were performed by Codex CoGenesis platform same as described in the somatic mutation calling and bioinformatics analysis above. A testing result report including the variants in genes and the variant allele frequency were submitted for assessment by EMQN.

### 2.9 Insert size evaluation

Read pairs supporting either mutant or non-mutant alleles of potentially clinically actionable genes, including *KRAS, TP53, PIK3CA, BRAF* and *EGFR* from the 16 patient plasma samples were identified using positional CIGAR-based alignment pattern matching for SNVs and indels. These reads were sorted into separate BAM files, and their insert size distributions were extracted using samtools stats. All sorted reads were combined to calculate their respective distributions and plot as a density distribution graph. Statistical significance of the insert size difference was assessed using a one-sided Wilcoxon rank sum test to determine whether mutant reads insert sizes are significantly shorter than those from the non-mutant reads.

## 3 Results

### 3.1 Comparison of liquid-liquid and direct solid-phase extraction using reference material

ctDNA Reference Material containing known mutations with 0.5% variant allele frequencies (VAFs) was used to assess and compare the performance of liquid-liquid (PHASIFY MAX) and direct solid-phase (QCNA) extraction kits with targeted panel sequencing. The reference material was comprised of a synthetic plasma solution and is commercially available for the purpose of validating sequencing-based assays and evaluating their performance in detecting genomic alterations at very low frequencies. TruSight Oncology 500 (TSO500) targeted panel was used to cover the 38 known DNA mutations in this Reference Material. DNA extracted from 1mL of the Reference Material was used for library preparation. The yield of DNA extracted from 1mL Reference Material was 19.6 ng and 14.0 ng for the liquid-liquid and solid-phase extraction systems, respectively. All of the extracted DNA was used for library preparation.

**Figure 1:**
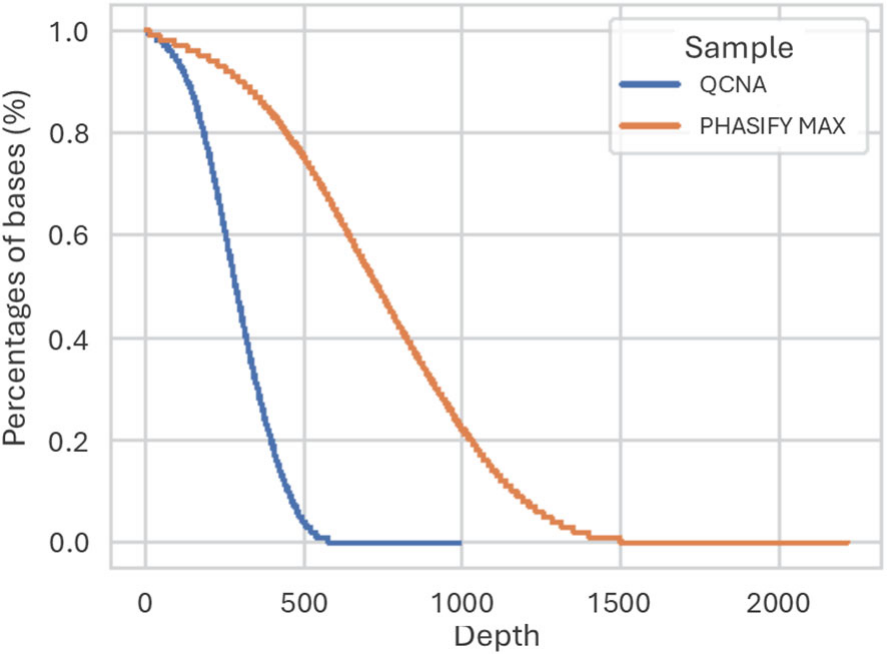
Sequencing depth of targeted bases from 1mL of reference material extracted by direct solid phase (QCNA) and liquid-liquid extraction (PHASIFY MAX). The y-axis represents the percentage of targeted bases that were able to achieve a given minimum sequencing depth, represented on the x-axis.

When evaluating the sequencing performance characteristics, the liquid-liquid extraction method had a higher percentage of aligned reads in target region relative to solid-phase extraction method (82.8% and 77.6%, respectively), as well as higher median unique fragment coverage on target bases (876x and 292x, respectively) (**Figure 1**; **Table 1A**). The number of variant calls was also measured and compared between the extraction methods. The liquid-liquid and solid-phase extraction methods demonstrated 94.7% (36/38) and 81.6% (31/38) detection rate in the 38 low frequency variant alleles, respectively. The comparison of the measured VAFs for each reference mutation is shown in **Table 1B**.

**Table 1a:** Comparison of the sequencing quality metrics using TSO500 panel from QCNA or PHASIFY MAX extracted reference material.

Extraction kit	QCNA	PHASIFY MAX
Total number of reads passing filter	120.8M	155.5M
Percentage of aligned reads in target region	77.6	82.8
Median unique fragment coverage on target bases	292	876
Percentage of target bases with greater than 100X unique fragment coverage	94.9	98.4
Percentage of target bases with greater than 250X unique fragment coverage	63.3	95.4
Coding region size in megabases	1.27	1.27
Total number of qualified variants for total TMB calculation	2	2
Total number of usable microsatellite (MSI) sites	104	123
Total number of unstable MSI sites	3	2

**Table 1b:** Comparison of VAFs detected in the reference materials when extracted using QCNA or PHASIFY MAX kit.

Gene	COSMIC	Theoretical AF%	QCNA	PHASIFY MAX
*AKT1*	COSM33765	0.525	N/A	0.24%
*APC*	COSM13127	0.510	0.66%	0.30%
*APC*	COSM18561	0.435	0.83%	0.53%
*ATM*	COSM21924	0.630	0.63%	0.59%
*BRAF*	COSM476	0.525	N/A	0.89%
*CTNNB1*	COSM5664	0.665	0.30%	0.28%
*EGFR*	COSM6224	0.615	0.79%	0.54%
*EGFR*	COSM12378	0.565	0.21%	0.69%
*EGFR*	COSM6225	0.580	0.64%	0.46%
*EGFR*	COSM6240	0.565	0.45%	0.72%
*ERBB2*	COSM20959	0.490	N/A	N/A
*FGFR3*	COSM715	0.550	N/A	0.46%
*FLT3*	COSM783	0.490	0.64%	0.40%
*FOXL2*	COSM33661	0.480	0.63%	0.44%
*GNA11*	COSM52969	0.690	0.79%	0.65%
*GNAQ*	COSM28758	0.490	0.38%	0.15%
*GNAS*	COSM27887	0.585	0.90%	0.69%
*IDH1*	COSM28747	0.665	N/A	0.25%
*JAK2*	COSM12600	0.500	N/A	0.31%
*KIT*	COSM1314	0.555	0.42%	0.49%
*KRAS*	COSM521	0.490	0.38%	0.31%
*MPL*	COSM18918	0.545	0.26%	0.31%
*NPM1*	COSM17559	0.395	N/A	N/A
*NRAS*	COSM584	0.625	0.40%	0.79%
*PDGFRA*	COSM736	0.565	0.33%	0.45%
*PDGFRA*	COSM28053	0.570	0.30%	0.38%
*PIK3CA*	COSM763	0.425	1.60%	0.89%
*PIK3CA*	COSM12464	0.460	1.05%	0.60%
*PIK3CA*	COSM775	0.555	0.33%	0.98%
*PTEN*	COSM4986	0.540	0.40%	1.06%
*PTEN*	COSM5809	0.540	1.20%	1.21%
*RET*	COSM965	0.535	1.22%	0.27%
*SMAD4*	COSM14105	0.500	0.70%	1.07%
*TP53*	COSM10648	0.570	0.61%	0.44%
*TP53*	COSM10660	0.525	1.23%	0.55%
*TP53*	COSM10662	0.530	0.20%	0.50%
*TP53*	COSM6530	0.470	0.21%	0.34%
*TP53*	COSM18610	Not tested	0.62%	0.45%

### 3.2 Extraction, library preparation, and sequencing characterization with clinical samples

To evaluate the feasibility of liquid biopsy-based tumor mutation detection using liquid-liquid cfDNA extraction, paired plasma and tumor tissue samples were collected from 16 CRC patients. The mean age of the patients was 63 years old (range: 44-85). All patients had tumor sizes of pT staging 3 or above, and 25% had pT staging 4. The demographic and tumor characterization information of the patient cohort is listed in **Supplementary Table 1**. Owing to the limited plasma volume available from biobanked clinical specimens, parallel DNA extraction using both liquid–liquid and direct solid−phase methods was not feasible. Instead, clinical samples were used to independently evaluate the performance of cancer−associated mutation detection in plasma DNA extracted by liquid–liquid extraction, with results assessed against matched tumor tissue. This analysis extends the initial validation performed with reference materials to a real−world setting. Plasma samples were extracted using PHASIFY MAX kit and tumor tissue samples were extracted using QIAGEN Allprep DNA/RNA mini kit. The DNA extraction characteristics are listed in **Supplementary Table 2a**. High variability in DNA recovery between patient samples was observed. DNA extracted from 2mL of plasma had a median recovery of 19.1 ng and ranged from 4.4 to 66.0 ng. Extraction yield was greater than 10 ng of plasma DNA in 14 out of 16 samples (87.5%). Twelve samples yielded less than the manufacturer recommended 40 ng input for TSO500. For each sample, up to 40 ng of extracted DNA was used for library preparation before target panel sequencing with Illumina TruSight Oncology 500. The average size of the extracted cfDNA peak from the 16 plasma samples was 161 bp (**Supplementary Table 2a**), while the average library size peak was 330 bp (**Supplementary Table 2b**). Despite the majority of samples being below the recommended input, all 16 plasma libraries were sequenced successfully, achieved Q30 scores greater than 85%. The sequencing process generated an average of 212.8 ± 45 million and 153.3 ± 63 million reads passing filter per plasma and tumor sample, respectively. Over 90% of regions have >100x coverage on average and the average median coverage at target region was 1,180 for plasma and 1,133 for tumor sample. Detailed sequencing output characteristics are shown in **Supplementary Table 3**.

### 3.3 Mutational profile comparison between tissue and liquid biopsies

**Figure 2:**
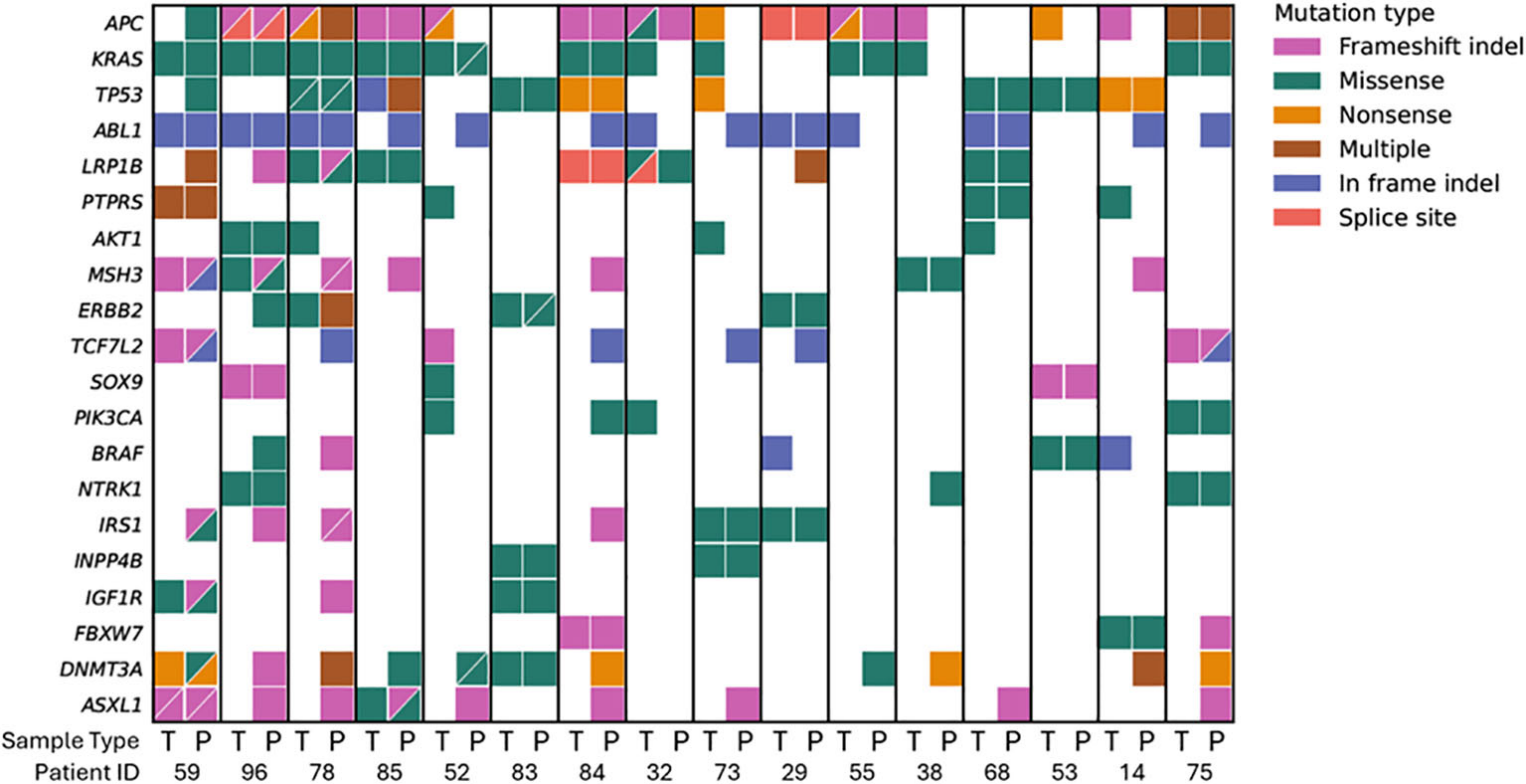
Mutation landscape of paired tumor tissue (T) and plasma (P) from each patient detected by TSO500 targeted sequencing. The top 20 most frequently mutated genes in tissue are shown. Mutation types are represented in pink (frameshift indel), green (missense), orange (nonsense), brown (multiple), blue (in frame indel), and red (splice site).

The TSO500 targeted panel was used to compare the mutational profiles between the different sample matrices, which detect 523 pan-cancer biomarkers with all variant types. The targeted sequencing approach used in this study encompassed a comprehensive panel of cancer-relevant genes, including *KRAS/NRAS* and *BRAF* mutation for CRC, in alignment with the National Comprehensive Cancer Network (NCCN) guidelines. Results of the mutation profiles of the top 20 mutated genes from the paired tissue and plasma samples are shown in **Figure 2**. The full list of somatic mutation calls in tissue and plasma samples can be found in **Supplementary Data Sheet 1**. *APC, KRAS* and *TP53* were the most commonly identified mutated genes in the 16-patient cohort for tumor tissue. These mutated genes were detected in 81.3%, 68.8% and 50% of tissue samples, respectively, and 56.3%, 50.0% and 50.0% of plasma samples, respectively.

The predominant mutation types varied across different genes. The most common mutation observed in *APC* is frameshift indel mutation, followed by nonsense mutation. *KRAS* mutations were limited to missense substitution, while *TP53* mutations were dominated by nonsense and missense mutations. Most of the variants observed in tumors were also observed in plasma. The positive detection rate for plasma detecting the top 3 mutations in tumor, *APC, KRAS* and *TP53*, were 61.5% (8/13), 72.7% (8/11) and 87.5% (7/8), respectively. Same type and point of mutation are detected in the paired tissue and plasma sample. For example, patients 75, 78, 84 and 85 were previously tested for *KRAS* mutation status using qPCR approach on tumor tissue samples and they are known to be positive for *KRAS* mutation (**Table 2**). Consistent with our sequencing result, *KRAS* c.35G>T mutation is detected in tumor sample of patient 75 and 84, while *KRAS* c.35G>A and *KRAS* c.437C>T are detected in tumor sample of patient 78 and 85, respectively, and the same specific mutation are detected in the plasma sample of the corresponding patients (**Table 2**). The matched mutation between plasma and tumor sample indicates that plasma can be used to reliably detect cancer mutation, especially when tumor tissue is unavailable. Lower mutation frequencies were observed in plasma compared to tumor tissue samples due to the dilution of ctDNA from the tumor tissue with non-tumor derived cfDNA from other parts of the body (18). The tumor mutation detection rate is defined as the percentage of number of mutations in tumor that is detected in plasma. The overall tumor mutation detection rate in plasma samples was 73.4% (262/357) (**Table 3**). Over 70% of the mutation which is not detected in plasma originated from subclonal mutation with low VAF (<0.25) in tumor sample. Therefore, the somatic mutation detection rate in plasma is lower when the VAF in tissue is low.

**Table 2:** Summary of *APC, KRAS, TP53* mutational profile of matched tumor and plasma samples detected on TSO500.

	*APC*		*KRAS*		*TP53*	
Patient ID	Mutation detected in tissue	Mutation detected in plasma	Mutation detected in tissue	Mutation detected in plasma	Mutation detected in tissue	Mutation detected in plasma
14	c.3736_3740del (MAF 33.3%)	Negative	Negative	Negative	c.637C>T (MAF 96.6%)	c.637C>T (MAF 0.7%)
29	c.834G>A (MAF 35.9%); c.1312+2T>C (MAF 42.4%)	c.834G>A (MAF 3.5%); c.1312+2T>C (MAF 6.3%)	Negative	Negative	Negative	Negative
32	c.4432_4460del (MAF 81.7%); c.6614C>G (MAF 41.1%)	c.4432_4460del (MAF 1.2%)	c.35G>T (MAF 38.5%)	Negative	Negative	Negative
38	c.4666dup (MAF 83.3%)	Negative	c.183A>C (MAF 45.6%)	Negative	Negative	Negative
52	c.1690C>T (MAF 16.3%); c.4476del (MAF 12.6%)	Negative	c.38G>A (MAF 24.7%)	c.38G>A (MAF 0.4%) c.404G>C (MAF 20%)	Negative	Negative
53	c.646C>T (MAF 66.9%)	Negative	Negative	Negative	c.1010G>T (MAF 64.4%)	c.1010G>T (MAF 1.1%)
55	c.2821G>T (MAF 19.4%); c.4364del (MAF 41.9%)	c.4364del (MAF 4.7%)	c.34G>A (MAF 61.9%)	c.34G>A (MAF 9.8%)	Negative	Negative
59	Negative	c.5463G>T (MAF 0.6%)	c.38G>A (MAF 11.9%)	c.38G>A (MAF 10.9%)	Negative	c.740A>G (MAF 0.5%)
68	Negative	Negative	Negative	Negative	c.817C>T (MAF 14.1%)	c.817C>T (MAF 5.9%)
73	c.2932C>T (MAF 77.7%)	Negative	c.35G>A (MAF 53.9%)	Negative	c.586C>T (MAF 79.1%)	Negative
75	c.1213C>T (MAF 52.7%); c.1409-1G>A (MAF 28.7%); c.4348C>T (MAF 55.6%)	c.1213C>T (MAF 9.7%); c.1409-1G>A (MAF 11.3%); c.4348C>T (MAF 9.0%)	c.35G>T (MAF 57.7%)	c.35G>T (MAF 17.2%)	Negative	Negative
78	c.3340C>T (MAF 42.1%); c.4215dup (MAF 43.1%)	c.3340C>T (MAF 19.5%); c.4215dup (MAF 20.7%); c.6747del (MAF 0.4%)	c.35G>A (MAF 57.8%)	c.35G>A (MAF 45.9%)	c.632C>T (MAF 45.7%); c.454C>T (MAF 44.4%)	c.632C>T (MAF 22.9%); c.454C>T (MAF 19.4%)
83	Negative	Negative	Negative	Negative	c.524G>A (MAF 35.0%)	c.524G>A (MAF 1.7%)
84	c.1387dup (MAF 78.3%)	c.1387dup (MAF 0.8%)	c.35G>T (MAF 61.8%)	c.35G>T (MAF 0.6%)	c.1024C>T (MAF 77.6%)	c.1024C>T (MAF 1.5%)
85	c.4064_4065insA (MAF 81.5%)	c.4064_4065insA (MAF 14.3%)	c.437C>T (MAF 79.2%)	c.437C>T (MAF 6.2%)	c.480_482del (MAF 80.1%)	c.480_482del (MAF 8.0%) c.761T>G (MAF 0.2%) c.743G>A (MAF 3.5%) c.451C>A (MAF 0.2%) c.379T>C (MAF 0.2%)
96	c.1548+1G>T (MAF 29.7%); c.4127_4128del (MAF 62.9%)	c.1548+1G>T (MAF 10%); c.4127_4128del (MAF 29.3%)	c.34G>A (MAF 44.6%)	c.34G>A (MAF 17%)	Negative	Negative

**Table 3:** Number of somatic mutations detected in tumor and plasma samples from each patient and the sensitivity of plasma to detect the mutation in tumor.

Patient ID	Number of mutations in tumor	Number of mutations in tumor that is detected in plasma	Tumor mutation detection rate of plasma
14	13	8	62% (8/13)
29	19	12	63% (12/19)
32	16	5	31% (5/16)
38	14	7	50% (7/14)
52	23	5	22% (5/23)
53	15	10	67% (10/15)
55	15	11	73% (11/15)
59	99	87	88% (87/99)
68	11	7	64% (7/11)
73	24	14	58% (14/24)
75	11	11	100% (11/11)
78	19	17	89% (17/19)
83	22	18	82% (18/22)
84	16	11	69% (11/16)
85	18	17	94% (17/18)
96	22	22	100% (22/22)
Overall	357	262	73.4% (262/357; 95% CI: 0.6857 to 0.7771)

### 3.4 Plasma can enhance the detection of potentially actionable genes

While some tumor mutations were not detected by liquid biopsy, we observed that mutations in potentially actionable genes for CRC were identified in plasma samples but not found in tumor tissue samples. Identification of mutations in potentially actionable genes is crucial to guide precision treatment strategies, and the combined use of plasma and tumor sample testing enhanced the positive detection of several potentially actionable target genes in the CRC patients. Specifically, two patients (patient ID 96 and 78) were found to harbor *BRAF* mutations which were missed by the tumor sample, increasing the positive detection rate of BRAF mutation from 19% with tissue sample alone to 31% with the combined plasma and tissue result (**Table 4**). Moreover, one patient (patient ID 84) was found to have a *PIK3CA* mutation which is not detected by the tumor sample, leading to the increase of *PIK3CA* mutation positive detection rate from 19% to 25% with combined plasma and tissue result. Similarly, two additional patients (patient ID 59 and 96) were found to have *EGFR* mutations that were not detected by the tumor sample, contributing to 13% of *EGFR* mutation rate with plasma compared to no detection of *EGFR* mutation with tissue sample alone. The absence of detection in the tumor tissue may be a result of tumor heterogenicity or the presence of other distal tumors (19). Therefore, plasma cfDNA analysis offers a complementary approach to tissue biopsies, capturing a more comprehensive analysis of the patient’s overall cancer mutation profile for treatment decision.

**Table 4:** Mutational profile of matched tumor and plasma samples for potentially actionable genes including *BRAF, PIK3CA, EGFR*.

Patient ID	Mutation detected in tissue	Mutation detected in plasma
14	*BRAF* c.95_100del (MAF 23.4%)	Not detected
29	*BRAF* c.95_100del (MAF 15.8%)	Not detected
32	*PIK3CA* c.1633G>A (MAF 40.3%)	Not detected
38	Not detected	Not detected
52	*PIK3CA* c.1633G>A (MAF 13.2%)	Not detected
53	*BRAF* c.94G>C (MAF 25%)	*BRAF* c.94G>C (MAF 11.8%)
55	Not detected	Not detected
59	Not detected	*EGFR* c.1159_1160del (MAF 0.3%)
68	Not detected	Not detected
73	Not detected	Not detected
75	*PIK3CA* c.3140A>G (MAF 50.0%)	*PIK3CA* c.3140A>G (MAF 12.0%)
78	Not detected	*BRAF* c.2257del (MAF 0.3%)
83	Not detected	Not detected
84	Not detected	*PIK3CA* c.1624G>A (MAF 0.6%)
85	Not detected	Not detected
96	Not detected	*BRAF* c.209G>A (MAF 9.6%); *EGFR* c.412A>G (MAF 0.5%)

### 3.5 Independent validation using blinded samples from EMQN

To further evaluate the robustness of using liquid-liquid extraction on plasma samples for target sequencing, an independent validation was performed using 3 blinded cancer plasma samples, provided by the European Molecular Genetics Quality Network (EMQN; Lab ID 3059). EGFR c.2582T>A p.(Leu861Gln) variant at 5.1% VAF was detected in case number 1. EGFR c.2369C>T p.(Thr790Met) variant at 2.8% VAF and c.2237_2245del p.(Glu746_ser752delinsAla) variant at 1.7% VAF were detected in case number 2, while KRAS c.34G>T p.(Gly12Cys) variant at 4.4% VAF was detected in case number 3. After submission of the sequencing results to EMQN, their report confirmed full concordance of identified mutations. In summary, this result supported the high accuracy and reliability of the PHASIFY liquid-liquid phase extraction and targeted sequencing combined workflow in an independent validation.

### 3.6 Length analysis for liquid biopsy plasma samples

**Figure 3:**
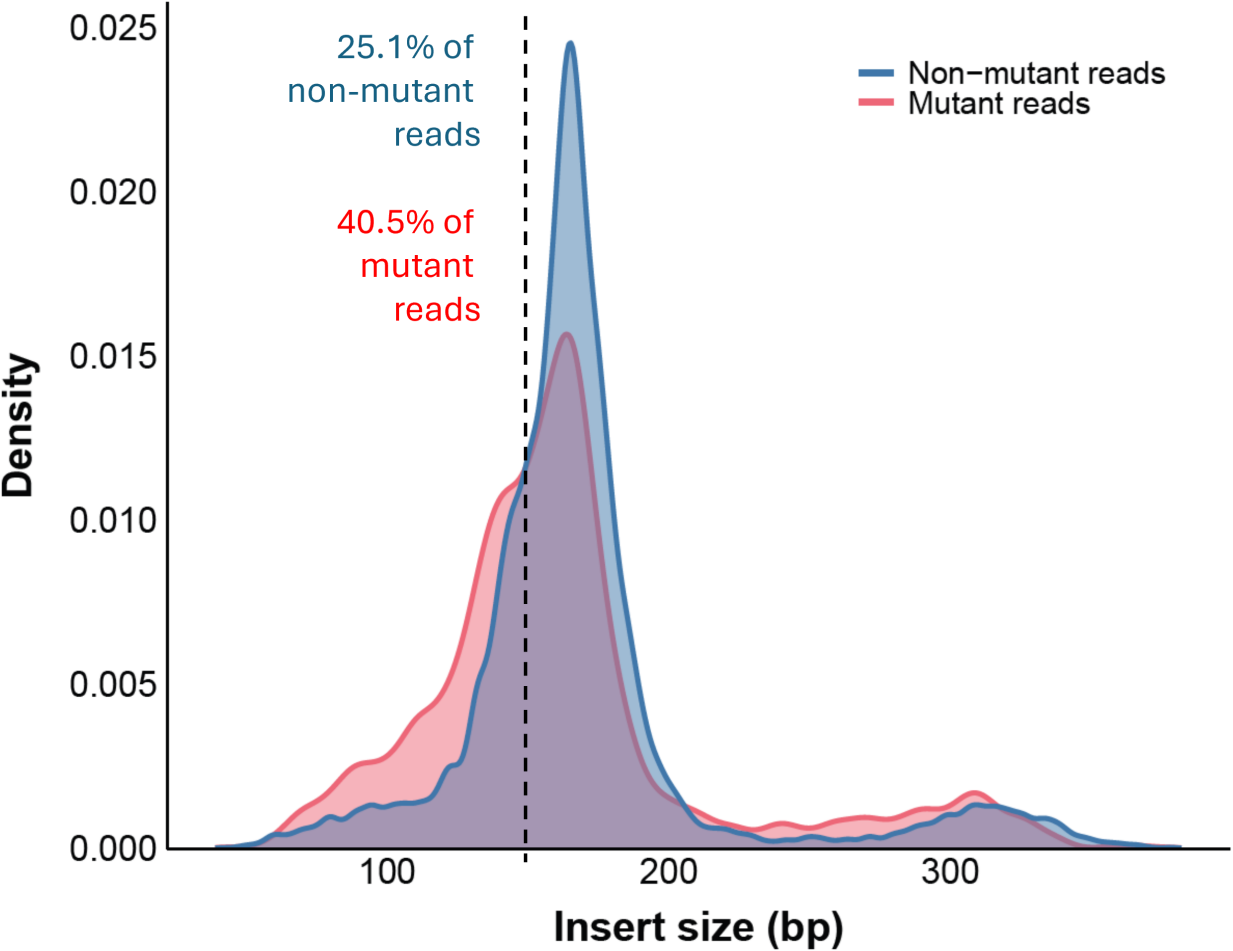
Plasma cfDNA size distribution for potentially clinically actionable genes from 16 CRC patients determined by sequencing. Reads carrying the *KRAS*, *TP53*, *PIK3CA*, *BRAF* and *EGFR* gene mutation are regarded as mutant reads. A dotted line indicates the location of 150bp insert size and percentage of reads shorter than 150bp are presented.

Analysis and comparison of the DNA fragment length between the ctDNA and the presumed healthy tissue cfDNA was performed using the insert size of the sequenced reads (**Figure 3**). To accomplish this, the reads with the *KRAS, TP53, PIK3CA, BRAF* and *EGFR* tumor-specific mutation were designated as tumor derived ctDNA, while the non-mutant reads with the wild-type sequence were designated as non-tumor derived cfDNA. It is noted that some of the presumed healthy tissue cfDNA will have also originated from tumor cells as the 16 patients did not express mutations in all 3 genes. We observed a global shift of the insert size profile toward shorter fragment sizes in the mutant reads compared to the non-mutant reads (**Figure 3**). Specifically, 40.5% of mutant reads (1149 out of 2839 reads) were below 150bp, in contrast to 25.1% of non-mutant reads (7942 out of 31681 reads). After further in silico segregation of mono-nucleosome and di-nucleosome fragments, grouped as 50 to 220bp and 221 to 400bp, respectively, we observed that the average insert sizes of the mutant reads are significantly smaller than the non-mutant reads in both mono-nucleosome (153bp vs 162bp, p=1.09E-84) and di-nucleosome (291bp vs 308bp, p=4.06E-15) region. The data suggests that ctDNA tends to have a shorter DNA fragment size compared to presumed healthy tissue cfDNA.

## 4 Discussion

Liquid biopsy offers a non-invasive alternative to tissue biopsies for cancer mutation detection, but the low abundance of ctDNA in plasma can limit sequencing sensitivity. In this study, analytical testing with reference materials demonstrated improved cfDNA yield, higher fragment coverages and higher tumor mutation detection rate with liquid-liquid extraction compared to a direct solid-phase extraction kit. These analytical observations suggest that the method can support downstream NGS analysis even when plasma input is limited.

The higher cfDNA yield and improved sequencing robustness may be a result of liquid-liquid extraction systems to concentrate and purify DNA. Standard direct solid-phase extraction systems operates by directly exposing a liquid biopsy sample to silica-based columns or magnetic beads which have an affinity for DNA, allowing the DNA to bind or adsorb to the surface, then removing the supernatant along with any contaminants, and finally eluting the DNA with a solution that disrupts the surface binding or adhesion. This process may be less efficient when target DNA concentrations are low or when contaminants compete for binding (20, 21). In contrast, liquid-liquid extraction systems concentrate DNA to a small volume with other potential contaminants removed prior to final recovery steps. The liquid-liquid extraction system used in this study was comprised of aqueous two-phase system (ATPS) components, which create distinct environmental differences between the two liquid phases that drive the partitioning of DNA to one phase, and protein and other contaminants to the opposite phase (22, 23). A potential concern for liquid-liquid phase extraction is that the substance carryovers during extraction can introduce PCR inhibitors for downstream applications. However, our previous report has successfully demonstrated the use of ATPS system in extracting plasma DNA for droplet digital PCR (ddPCR) as downstream diagnostic tool (12). The application of ATPS system has also extended to urine DNA isolation for cervical HPV qPCR test (24), further showing the well compatibility of ATPS system to PCR.

Although the clinical sample evaluation did not include a head−to−head comparison with a direct solid−phase extraction kit, the performance of the liquid–liquid extraction workflow was evaluated against matched tumor tissue using clinical samples. This evaluation allows better understanding of the liquid-liquid extraction protocol under real−world clinical conditions, where extracted ctDNA amount can vary substantially between patients. Using the liquid–liquid method, DNA extraction from 2 mL CRC plasma samples yielded a median of 19.1 ng. Previous report showed that cfDNA yield in plasma can vary between 5 to 1,142 ng, while cancer stage, cancer type, ctDNA clearance rate and plasma volume were major factors determining the ctDNA yield (25).

Previous clinical validation studies have demonstrated that lower DNA input can increase the risk of sequencing quality control failures and incomplete variant detection (26). In fact, the TruSight Oncology 500 assay recommended using 40 ng DNA input for optimal performance. However, in real−world clinical settings, achieving a 40 ng input may be challenging, particularly for patients with early-stage cancer, cancer type with low ctDNA shedding, high ctDNA clearance rate, or limited plasma volume.

Although the reduced DNA input in this study was expected to compromise detection sensitivity, the liquid–liquid extraction workflow combined with targeted NGS achieved mutation detection rates within or exceeding those reported in prior plasma studies that used solid−phase extraction methods with comparable or larger plasma input volumes (2–4 mL) (10). The average tumor mutation detection rate in plasma from these studies was 50% (95% credible interval (CI): 29-72%). There are some limitations to this comparison as the studies in this meta-analysis are whole exome target sequencing while our study is using a target gene panel of 523 genes. Despite this, a separate study in colorectal cancer patients, which used a 605-gene panel to assess mutation detection in paired plasma and tissue samples, reported a 49.2% (249 out of 506 variants detected in tissue) tumor mutation detection rate in plasma extracted by a direct solid phase system even at a higher sequencing depth (≥1,500x) than this study (27). Furthermore, another study also with colorectal cancer patients, that used 556 or 105-gene panels to detect the mutations in paired plasma and tumor tissue samples, demonstrated a 72.3% tumor mutation detection rate in plasma extracted by direct solid phase system, but at an ultra-deep sequencing depth of ≥7000x (28). These observations suggest that the liquid-liquid extraction approach performs adequately under lower-input conditions within this pilot study, although direct comparisons between liquid-liquid extraction and direct solid-phase extraction within clinical samples are needed to confirm this. Cross−study comparisons should nevertheless be interpreted with caution due to differences in targeted panels, sequencing depths, and analytical workflows. The validated results of the blinded worldwide external quality assessment scheme support the analytical accuracy of the workflow and provide independent confirmation of its performance on liquid biopsy-based oncology testing.

In our cohort, several potentially actionable gene mutations were detected in plasma but not in the corresponding tumor tissue. Three of the four patients (patients 78, 84, and 96) with mutations detected only in plasma had metastatic conditions, supporting the likelihood that these mutations originated from unsampled or distal tumor sites that contributed ctDNA to circulation. The remaining case (patient 59) exhibited mucinous histology, a subtype characterized by large extracellular mucin pools with sparse strips of tumor cells floating within the mucin, which is known to result in low tumor cellularity and regional heterogeneity in the tumor. Since tissue biopsy relies on a limited tumor section, it may not capture the mutations in spatially distinct tumor regions or low cellularity subclonal regions, while ctDNA from different regions could also shed into circulation and detected in plasma. The results demonstrated that parallel analysis of plasma and tumor samples may offer a more comprehensive evaluation on the patient’s genomic landscape to better guide treatment plans (29, 30).

Several factors correlate with the tumor mutation detection rate of plasma in different patients. First, metastatic disease (M1 status) was associated with markedly higher plasma tumor−mutation detection rates, consistent with the expectation that patients with advanced disease have greater ctDNA shedding from both primary and metastatic sites, while non−metastatic patients generally exhibited lower average sensitivity, although this was not universal. For example, the only two patients with tumor mutation detection rate below 40% are both non−metastatic cases. Additionally, for the mutations which are detected in tumor, the low tumor tissue variant allele frequency (VAF) have low plasma detectability, with the majority of mutations missed in plasma corresponding to tissue VAFs below 0.25. Meanwhile, plasma DNA input to library preparation also associated with the plasma tumor mutation detection rate. Samples with high input (40ng) consistently achieved high detection rate (89-100%), while sample with very low input (<10ng) showed relatively low detection rate (31-63%).

During extraction and sequencing characterization, we observed shorter DNA lengths in mutant reads compared to non-mutant reads, which corroborates the findings of previous studies on the shorter length pattern of ctDNA potentially contributed by differences in nucleosome wrapping and apoptotic fragmentation process (31, 32). This pattern suggests the potential to increase the mutation detection rate by enriching the ctDNA based on size, such as selectively recover fragments below 150bp. This could be particularly beneficial in enabling early-stage cancer detection using plasma samples and should be investigated further in future studies.

Notably, when compared with other previous colorectal cancer studies which used smaller panels ranging from 10 to 170 of gene target (33–35), the large panel size of 523 cancer-related genes in this study enabled the identification of a broader range of mutations and provided a more comprehensive evaluation when using plasma to detect the tumor-derived mutations. The large gene panel used in this study also enabled us to detect mutations that have been overlooked by previous studies using smaller gene panels. For example, mutations on *ABL1, LRP1B, NTRK1, IRS1* and *IGF1R* were detected in at least 2 out of the 16 patients and are not covered by smaller gene panels. These genes may play a role in regulating colorectal cancers and may warrant further investigation regarding their biological or clinical relevance. For example, *ABL1* mutation is associated with colorectal cancer progression through TGF-β and PI3K/Akt pathways (36), and LRP1B mutation can be used as a predictive biomarker for immune checkpoint inhibitor therapy (37, 38).

There are some limitations in this pilot study that should be acknowledged. Firstly, only one solid phase extraction kit was used for comparison during the analytical analysis with the liquid-liquid extraction. While the QCNA kit has been widely used for cfDNA extraction in both research and clinical setting (39–41), future studies should include a broader variety of extraction kits. Secondly, in this pilot cohort, only 2 mL of plasma was available per patient from the biobanked clinical samples, which limit the feasibility of performing parallel extractions on two extractions kit. Therefore, this pilot clinical study was designed to evaluate the feasibility and performance of the liquid-liquid extraction by using tumor tissue as the reference standard and determining the percentage of tumor mutation detectable in plasma. Parallel extraction on two extraction kits to directly compare the performance between the two was not achieved in this pilot clinical study. Lastly, the clinical cohort of 16 colorectal cancer patients represents an exploratory pilot sample size which limits the generalizability of the clinical findings. Although the successful blinded evaluation of EMQN materials provided additional confidence in the performance of the liquid-liquid plasma extraction and targeted panel next-generation sequencing integrated testing workflow, larger-scale clinical validation is required to fully establish its diagnostic utility in clinical oncology settings.

## 5 Conclusion

This pilot study demonstrates that, in analytical testing using reference materials, the liquid-liquid extraction platform shows improved sensitivity for detecting low-frequency variants compared to a commonly used solid-phase extraction kit. The clinical pilot study further establishes the feasibility of liquid-liquid based extracted ctDNA for downstream targeted sequencing for variant calling for cancer patient plasma samples. To our knowledge, this is among the first demonstration of applying liquid-liquid based extraction of plasma ctDNA for NGS-based analysis. This approach may advance liquid biopsy-based oncology testing by improving mutation detection with minimal blood draw.

## Data Availability

The original contributions presented in the study are publicly available. This data can be found here: the CNCB database (China National Center for Bioinformation); PRJCA062746.
