# Supplementary material for: A novel liquid-liquid cfDNA extraction method for targeted sequencing with colorectal cancer patient samples: a pilot study
Source: Front Oncol. 2026 Apr 29;16:1812135. doi: 10.3389/fonc.2026.1812135 (PMC13167482; doi:10.3389/fonc.2026.1812135)
Supplement: Supplementary file 2 [file Table1.docx]

Supplementary Material

# Supplementary Figures and Tables

| **Patient ID** | **Gender** | **Age** | **Past Medical Health** | **Diagnosis** | **TNM/Dukes stage** | **Histologic type** | **Degree of differentiation** | **KRAS mutation status, qPCR, tissue** | **Adenoma/Polyps** |  |
| --- | --- | --- | --- | --- | --- | --- | --- | --- | --- | --- |
|  |  |  |  |  |  |  |  |  |  |  |
| 14 | M | 85 | DM, HT, CVA, renal impairment | adenocarcinoma | T3N1M0/C | adenocarcinoma | moderate | unknown | present |  |
|  |  |  |  |  |  |  | differentiated |  |  |  |
| 29 | M | 50 | Good | adenocarcinoma | T3N0M0/B | adenocarcinoma | moderate | unknown | present |  |
|  |  |  |  |  |  |  | differentiated |  |  |  |
| 32 | M | 62 | HT, asthma | adenocarcinoma | T3N1M0/C | adenocarcinoma | moderate | unknown | present |  |
|  |  |  |  |  |  |  | differentiated |  |  |  |
| 38 | F | 76 | HT, Hep B carrier | adenocarcinoma | T3N1M0/C | adenocarcinoma | moderate | unknown | present |  |
|  |  |  |  |  |  |  | differentiated |  |  |  |
| 52 | F | 63 | HT | adenocarcinoma | T4N1M0/C | adenocarcinoma | moderate | unknown | present |  |
|  |  |  |  |  |  |  | differentiated |  |  |  |
| 53 | M | 63 | DM, HT | adenocarcinoma | T4N0M0/B | adenocarcinoma | moderate | unknown | present |  |
|  |  |  |  |  |  |  | differentiated |  |  |  |
| 55 | M | 70 | DM, HT, CKD | adenocarcinoma | T3N2M0/C | adenocarcinoma | moderate | unknown | present |  |
|  |  |  |  |  |  |  | differentiated |  |  |  |
| 59 | F | 74 | HT | adenocarcinoma | T3N1M0/C | adenocarcinoma | mucinous | unknown | absence |  |
| 68 | F | 59 | Good | adenocarcinoma | T3N0M1/D | adenocarcinoma | moderate | unknown | absence |  |
|  |  |  |  |  |  |  | differentiated |  |  |  |
| 73 | F | 58 | Good | adenocarcinoma | T2N0M0/B | adenocarcinoma | moderate | unknown | present |  |
|  |  |  |  |  |  |  | differentiated |  |  |  |
| 75 | F | 44 | Good | adenocarcinoma | T4N0M1/D | adenocarcinoma | moderate | positive | absence |  |
|  |  |  |  |  |  |  | differentiated |  |  |  |
| 78 | M | 68 | CVA | adenocarcinoma | T3N0M1/D | adenocarcinoma | moderate | positive | absence |  |
|  |  |  |  |  |  |  | differentiated |  |  |  |
| 83 | M | 65 | HT, hyperlipidaemia | adenocarcinoma | T3N0M0/B | adenocarcinoma | moderate | unknown | unknown |  |
|  |  |  |  |  |  |  | differentiated |  |  |  |
| 84 | M | 63 | HT, hyperlipidaemia | adenocarcinoma | T3N0M1/D | adenocarcinoma | moderate | positive | unknown |  |
|  |  |  |  |  |  |  | differentiated |  |  |  |
| 85 | M | 78 | HT, hyperlipidaemia | adenocarcinoma | T4N2M1/D | adenocarcinoma | moderate | positive | present |  |
|  |  |  |  |  |  |  | differentiated |  |  |  |
| 96 | F | 57 | Good | adenocarcinoma | T3N0M1/D | adenocarcinoma | N/A | unknown | unknown |  |

**Supplementary Table 1.** Baseline demographics and clinicopathological characteristics of patients.

|  | | **Patient ID** | | | | | | | | | | | | | | | | |
| --- | --- | --- | --- | --- | --- | --- | --- | --- | --- | --- | --- | --- | --- | --- | --- | --- | --- | --- |
|  |  | **52** | **53** | **75** | **85** | **73** | **83** | **84** | **14** | **29** | **32** | **38** | **55** | **59** | **68** | **78** | **96** |  |
| **Plasma sample** | **Extraction kit:** | **PHASIFY Plasma MAX 2.0** | | | | | | | | | | | | | | | | |
|  | **DNA Quantity (ng):** | 21.8 | 18.4 | 66.0 | 43.2 | 19.0 | 14.0 | 31.8 | 12.5 | 9.8 | 4.4 | 18.4 | 13.3 | 24.1 | 19.3 | 54.3 | 49.0 |  |
|  | **cfDNA peak size (bp)** | 176 | 173 | 163 | 177 | 128 | 109 | 131 | 177 | 176 | 181 | 171 | 178 | 177 | 155 | 155 | 156 |  |
| **Tumor sample** | **Extraction kit:** | **Qiagen Allprep DNA/RNA mini kit** | | | | | | | | | | | | | | | | |
|  | **DNA Quantity (ng):** | 1930 | 5400 | 4360 | 3450 | 2150 | 9840 | 4660 | 3370 | 3280 | 2040 | 3310 | 4710 | 2250 | 2090 | 5000 | 6690 |  |

**Supplementary Table 2a.** DNA concentration summary of each paired sample extracted

|  | | **Patient ID** | | | | | | | | | | | | | | | |
| --- | --- | --- | --- | --- | --- | --- | --- | --- | --- | --- | --- | --- | --- | --- | --- | --- | --- |
|  | | **52** | **53** | **75** | **85** | **73** | **83** | **84** | **14** | **29** | **32** | **38** | **55** | **59** | **68** | **78** | **96** |
|  | **Library size of plasma sample (bp):** | 337 | 335 | 332 | 334 | 330 | 333 | 334 | 328 | 328 | 327 | 324 | 333 | 330 | 333 | 330 | 325 |
|  | **Library size of tumor sample (bp):** | 404 | 400 | 410 | 390 | 348 | 361 | 355 | 352 | 356 | 352 | 370 | 364 | 367 | 355 | 343 | 344 |

**Supplementary Table 2b.** Characteristics of final libraries created from each paired sample.

| **Patient ID:** | **52** | **53** | **75** | **85** | **73** | **83** | **84** | **14** | **29** | **32** | **38** | **55** | **59** | **68** | **78** | **96** |
| --- | --- | --- | --- | --- | --- | --- | --- | --- | --- | --- | --- | --- | --- | --- | --- | --- |
| **Total number of reads passing filter** | 242525842 | 222133556 | 259211464 | 264443858 | 214894242 | 193877372 | 126050748 | 142191912 | 151494562 | 183624202 | 234780392 | 177140308 | 260306774 | 232825878 | 249159300 | 249545478 |
| **Raw sequencing coverage** | 12501 | 11450 | 13361 | 13631 | 16616 | 14991 | 9746 | 10994 | 11713 | 14198 | 18153 | 13696 | 20127 | 18002 | 19265 | 19295 |
| **Median fragment coverage of target bases** | 1318 | 1033 | 1955 | 1626 | 1052 | 515 | 1403 | 795 | 795 | 186 | 809 | 641 | 1227 | 1434 | 2306 | 1792 |
| **Percent target bases with greater than 100X coverage** | 91.9 | 90.3 | 99.3 | 96.4 | 98.7 | 98.4 | 98.9 | 94.2 | 97.5 | 67.2 | 90.5 | 96.6 | 97.7 | 99 | 99.5 | 99.1 |
| **Percent target bases with greater than 250X coverage** | 85.5 | 82.8 | 97.4 | 92.5 | 97.5 | 93.2 | 97.6 | 89.1 | 94 | 30.6 | 85.2 | 91.6 | 95.7 | 98.2 | 98.9 | 98.5 |
| **Coding region size in megabases** | 1.21 | 1.19 | 1.28 | 1.25 | 1.28 | 1.27 | 1.28 | 1.25 | 1.27 | 1.02 | 1.22 | 1.27 | 1.27 | 1.28 | 1.28 | 1.28 |
| **Total number of qualified variants for total TMB calculation** | 4 | 2 | 4 | 11 | 3 | 3 | 4 | 1 | 4 | 1 | 1 | 3 | 58 | 5 | 14 | 13 |
| **Total number of usable microsatellite (MSI) sites** | 105 | 100 | 124 | 116 | 125 | 125 | 125 | 113 | 119 | 45 | 105 | 118 | 119 | 125 | 125 | 125 |
| **Total number of unstable MSI sites** | 0 | 2 | 1 | 3 | 3 | 3 | 2 | 5 | 4 | 2 | 1 | 2 | 8 | 5 | 4 | 6 |

**Supplementary Table 3a.** Complete profile of TSO500 sequencing metrics of plasma sample from each patient generated by illumina DRAGEN pipeline

| **Patient ID:** | **52** | **53** | **75** | **85** | **73** | **83** | **84** | **14** | **29** | **32** | **38** | **55** | **59** | **68** | **78** | **96** |
| --- | --- | --- | --- | --- | --- | --- | --- | --- | --- | --- | --- | --- | --- | --- | --- | --- |
| **Total number of reads passing filter** | 61413642 | 81591690 | 61080734 | 87331448 | 273756924 | 170236418 | 209751280 | 204846584 | 157764328 | 199772486 | 231167366 | 202274374 | 116712770 | 126024722 | 144107730 | 125512750 |
| **Raw sequencing coverage** | 3232 | 4294 | 3215 | 4596 | 14111 | 8775 | 10812 | 10559 | 8132 | 10298 | 11916 | 10427 | 9024 | 9744 | 11142 | 9705 |
| **Median coverage of target bases** | 412 | 449 | 239 | 468 | 1061 | 1190 | 1573 | 1524 | 1350 | 1456 | 1207 | 1627 | 1137 | 1642 | 1206 | 1594 |
| **Percent target bases with greater than 100X coverage** | 98.6 | 98.6 | 97.4 | 99 | 99.2 | 99.3 | 99.3 | 99.4 | 99 | 99.4 | 99.2 | 99.4 | 98.7 | 98.9 | 98.9 | 98.8 |
| **Percent target bases with greater than 250X coverage** | 77.2 | 76.1 | 76.8 | 76 | 98.4 | 98.4 | 98.4 | 98.6 | 98.1 | 98.7 | 98.3 | 98.6 | 97.3 | 97.9 | 97.9 | 97.9 |
| **Coding region size in megabases** | 1.27 | 1.27 | 1.27 | 1.28 | 1.28 | 1.28 | 1.28 | 1.28 | 1.28 | 1.28 | 1.28 | 1.28 | 1.28 | 1.28 | 1.28 | 1.28 |
| **Total number of qualified variants for total TMB calculation** | 14 | 7 | 3 | 16 | 13 | 14 | 14 | 5 | 12 | 14 | 10 | 11 | 90 | 10 | 16 | 16 |
| **Total number of usable microsatellite (MSI) sites** | 124 | 121 | 117 | 122 | 125 | 125 | 125 | 124 | 124 | 124 | 125 | 125 | 124 | 125 | 125 | 124 |
| **Total number of unstable MSI sites** | 0 | 1 | 1 | 4 | 3 | 5 | 6 | 5 | 6 | 1 | 2 | 4 | 47 | 3 | 4 | 5 |

**Supplementary Table 3b.** Complete profile of TSO500 sequencing metrics of tumor tissue sample from each patient generated by illumina DRAGEN pipeline
